# Rapid Determination of the Oil and Moisture Contents in *Camellia* *gauchowensis* Chang and *Camellia* *semiserrata* Chi Seeds Kernels by Near-infrared Reflectance Spectroscopy

**DOI:** 10.3390/molecules23092332

**Published:** 2018-09-12

**Authors:** Yingzhong Zhang, Liangbo Zhang, Jing Wang, Xuxiao Tang, Hong Wu, Minghuai Wang, Wu Zeng, Qihui Mo, Yongquan Li, Jianwei Li, Yijuan Huang, Baohua Xu, Mengyu Zhang

**Affiliations:** 1 Guangdong Provincial Key Laboratory of Silviculture, Protection and Utilization, Guangdong Academy of Forestry, Guangzhou 510520, China; zyzh3762@sinogaf.cn (Y.Z.); tangxuxiao666@163.com (X.T.); wangmh@sinogaf.cn (M.W.); baohuaxu@sinogaf.cn (B.X.); rainbow84397520@163.com (M.Z.); 2 Institute of Bioresource and Bioenergy, Hunan Academy of Forestry, Changsha 410004, China; zhangliangbo2001@aliyun.com (L.Z.); wuhong432@sina.com (H.W.); 3 Department of Science and Technology, Gaozhou Institute of Forestry, Maoming 525200, China; gdgzlks@163.com (W.Z.); 13828616962@139.com (J.L.); 4 Department of Science and Technology, Guangning Institute of Forestry, Zhaoqing 526300, China; 13536979935@163.com (Q.M.); hyj6762@163.com (Y.H.); 5 Department of Science and Technology, Guangdong Province Forestry Science and Technology Extension Station, Guangzhou 510173, China; gdf_lyq@gd.gov.cn

**Keywords:** near infrared reflectance spectroscopy, *Camellia* seeds kernel, oil content, moisture content

## Abstract

A fast and effective determination method of different species of vegetable seeds oil is vital in the plant oil industry. The near-infrared reflectance spectroscopy (NIRS) method was developed in this study to analyze the oil and moisture contents of *Camellia* *gauchowensis* Chang and *C.* *semiserrata* Chi seeds kernels. Calibration and validation models were established using principal component analysis (PCA) and partial least squares (PLS) regression methods. In the prediction models of NIRS, the levels of accuracy obtained were sufficient for *C.* *gauchowensis* Chang and *C.* *semiserrata* Chi, the correlation coefficients of which for oil were 0.98 and 0.95, respectively, and those for moisture were 0.92 and 0.89, respectively. The near infrared spectrum of crush seeds kernels was more precise compared to intact kernels. Based on the calibration models of the two *Camellia* species, the NIRS predictive oil contents of *C.* *gauchowensis* Chang and *C.* *semiserrata* Chi seeds kernels were 48.71 ± 8.94% and 58.37 ± 7.39%, and the NIRS predictive moisture contents were 4.39 ± 1.08% and 3.49 ± 0.71%, respectively. The NIRS technique could determine successfully the oil and moisture contents of *C.* *gauchowensis* Chang and *C.* *semiserrata* Chi seeds kernels.

## 1. Instruction

*Camellia*, native to China, one of the most important sources of high-quality edible plant oil, has been consumed for more than 1000 years in southern China [1] and is widely distributed with more than 12 million acres in production [2]. *Camellia gauchowensis* H.T. Chang and *Camellia semiserrata* C.W. Chi [3], two common woody species of *Camellia* indigenous to China, are mainly cultivated in mountain area. As cooking oil that has high nutritional value for human health, the annual *Camellia* oil production exceeds 150 million kilograms [4,5]. It has 85–92% unsaturated fatty acids [5] including 75–85% oleic acid that plays important roles in reducing cholesterol and triglycerides in the blood [6,7], and a variety of other functional components such as vitamin E, phytosterols, squalene, and flavonoids [8].

For the proper utilization of *Camellia* oils in food and other industries, the quality characteristics of *Camellia* seeds, such as the contents of oil, moisture and protein, and the composition of fatty acids, should be evaluated quickly and reliably by analytical tools at harvest, and during marketing and processing. Conventional analysis for oil and moisture are time-consuming, laborious, and require the use of toxic chemicals and expensive equipment [9]. The development of fast and effective determination methods is necessary, especially when the application needs are considered.

In recent years, there is a growing interest in fast, reliable and environmentally friendly technologies in both food production and food research [10]. Near-infrared reflectance spectroscopy (NIRS) technology is a simple and non-destructive method that can measure the quality and compositional attributes of many substances [11,12]. NIRS has some important advantages, such as short analysis time, minimal sampling process, and non-destructiveness, with a performance comparable to chemistry analytical methods [13,14]. NIRS is a spectroscopy method that uses the near-infrared region (400–2500 nm) of the electromagnetic spectrum [15] that can quantify organic compounds by the absorption of near-infrared light with the chemical bonds. Reflectance signatures are collected from sample materials with known nutrient concentrations, and then mathematical models are developed to estimate nutrient constituents of materials with unknown levels [16]. Automatic data collection by NIRS allows the assessment of multiple traits in large sets of samples [14] with short duration and with minimal error [16].

Based on the resolution of the analytical and quality factors from food samples with correlation of electromagnetic absorption at aforementioned wavelength, NIRS is used routinely in sensory, physical and chemical analysis of food and agricultural products [17,18,19]. As reported, it has been widely used in different crops, such as sesame [20], maize [21], rice [22], soybean [23] and sunflower [24], to predict oil, moisture, protein, fatty acids, phenols or crude fiber. However, the ability of NIRS for prediction of *Camellia* oil and moisture content, especially *C. gauchowensis* Chang and *C. semiserrata* chi, has not been reported.

It is necessary to rapidly determine oil and moisture contents in *Camellia* seeds, which play a key role in national production, importation as well as in food processing and breeding programs. Since analyses based on NIRS do not require labor intensive sample pre-treatment and processing, samples are measured with simple grinding or as a whole. In this study, we established and calibrated the quantitative NIRS models by principal component analysis (PCA) and partial least squares (PLS) for *C. gauchowensis* Chang and *C. semiserrata* Chi seeds kernels to quickly determine oil and moisture contents.

## 2. Materials and Methods

### 2.1. Materials

Each of 110 samples (200 g per sample) of *Camellia gauchowensis* Chang and *Camellia semiserrata* Chi seeds was obtained from the seed resource bank of Guangdong Academy of Forestry (Guangzhou, China). To guarantee the reliability and applicability of the models, samples were selected from different regions in Guangdong province such as Gaozhou, Huizhou, Yangjiang, Lianping, and Zhaoqing to ensure a wide range of reference values. When *Camellia* ripened from green to dark brown with the full maturity of peel split and seeds exposed, the seeds were collected and naturally air-dried. The inner shells of seeds were peeled off by tool knocking and manual peeling to get the *Camellia* kernels. The intact *Camellia* kernels were selected and preserved in a ventilated and dry place at room temperature. The period from the beginning of seed collection to the end of measurement was six months.

### 2.2. Soxhlet Extraction and Oven Drying

Traditional chemical measurements for oil and moisture were Soxhlet extraction [7] and oven drying [25], respectively. All kernels were powdered by a laboratory grinder and screened by 40-mesh. In the oil determination, samples were weighed as *W*_0_ (g), and then extracted at 80 °C for 6 h by Soxhlet extractor with 180 mL petroleum ether. The residues were dried at 60 °C in a vacuum oven to the constant weight as *W*_1_ (g). The oil content was calculated as C_1_ = *W*_1_/*W*_0_ × 100%. In the moisture measurement, the samples were weighted as *M*_0_ (g), and then dried at 105 °C in vacuum oven over 24 h until the weight was constant as *M*_1_. The moisture content was expressed as C_2_ = *M*_1_/*M*_0_ × 100%.

### 2.3. Sample Pretreatment

The *Camellia* seeds kernels were openly placed in an air-conditioned thermostat room at 20 ± 2 °C for two weeks to keep their moisture content under 10%. The kernels were loaded into a small round plastic cup (75 mm diameter and 100 mm height). It was ensured that all tested samples were representative and filled the sample tray uniformly. One batch of specimen was directly used for spectroscopic analysis and one batch of comminution was used for spectral decomposition.

### 2.4. Spectra Measurements

#### 2.4.1. Near Infrared Spectroscopy

A DA7200 NIRS analyzer (Perten Instruments AB, Huddinge, Sweden) equipped with simultaneous detection of two beams of halogen and mercury lamp was used for spectral measurement. Fixed holographic grating partial light and indium arsenic diode array detection technology with electric refrigeration constant temperature, full spectrum was simultaneously scanned. Spectral data were acquired in the 950–1650 nm range with 5 nm resolution at the collection data of 100 times per second. Simplicity software was integrated as a device manager.

#### 2.4.2. Spectral Feature Data Acquisition

The NIRS instrument was turned on to preheat for 1 h. Before the spectrum collection, RE-SULT-Integration software was used to compile spectrum acquisition program. The working parameters of the instrument were set as the measuring wavelength range from 950 to 1650 nm at 30 subsequent scanning times. The sampling mode was transmittance and the data acquisition was absorbance.

After writing the collection program, the processed samples were placed on the spectrometer sample tray in turn to be scanned. The near infrared spectra of samples were collected by the cup light probe. To reduce the error caused by inhomogeneous loading, each tested sample was loaded in duplicate and scanned in triplicate for each loading to obtain the spectrum from different angles, and average data were taken. All spectral characteristics were collected and recorded.

#### 2.4.3. Calibration and Validation Model Construction

The average spectra of each sample were collected for smoothing pretreatment by Unscrambler classifier chemometrics software package (CAMO, Trondheim, Norway). After correlating the reference analytical methods, the best calibration model was gained from several mathematical models. In this study, principal component analysis (PCA) [26] and partial least squares (PLS) [27] methods were finally used to construct calibration and validation models. The correlation coefficient of calibration (Rc), the standard error of calibration (SEC), standard error of prediction (SEP), offset, bias, slop, and the residual predictive value (RPD) were used to test the accuracy of the calibration models. Rc was gained from the calibration process that can be modeled linearly [7,28]. SEC and SEP were to evaluate the predictive ability of the calibration model [7,28,29]. Offset, bias, slop, and RPD were used to determine precision of the calibration [10,28]. The ratio of standard deviation (SD) and SEP was named RPD [30].

## 3. Results and Discussion

### 3.1. Comparison of Camellia Seeds Spectrograms between Non-Destruction and Comminution

**Figure 1:**
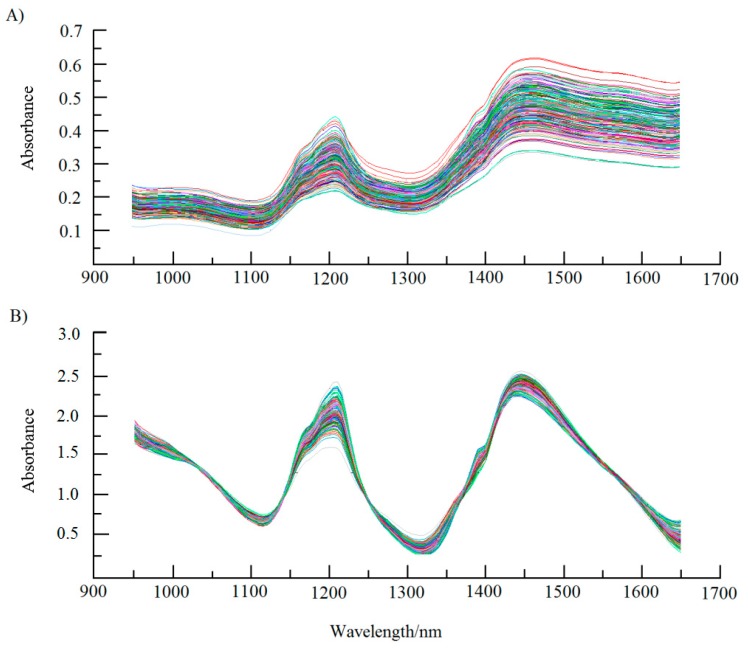
Near infrared spectrogram comparison of *Camellia gauchowensis* Chang seeds kernels between: (**A**) non-destruction; and (**B**) comminution. 110 *C*. *gauchowensis* Chang samples represeneted 110 color lines, the same as *C. semiserrata* Chi seeds kernels (Figure not shown).

NIRS is one kind of electromagnetic waves between visible and infrared light region, and its wavelength range is 400–2500 nm [15]. Different substances have different absorption and strength of electromagnetic waves [7]. The overall spectra of *Camellia* seeds kernels in different treatment showed strong absorption bands related with oil and water content. The absorption at around 1200 nm (C–H) is related with oil [26] and at 1450 nm (O–H) with moisture [31]. The raw NIR spectra of non-destruction and comminution of *Camellia* samples are shown in Figure 1. Compared with Figure 1A, the crushed kernels showed relatively wide range of absorbance over the entire NIR spectra in Figure 1B with a sharper upward and downward slopes in absorbance around 1200 and 1450 nm, respectively. The near-infrared reflectance spectrogram (NIRS) of *Camellia* seeds kernels could be used for the quantitative analysis and the selection of suitable sample types. In addition, the NIRS accuracy was higher when the *Camellia* seeds kernels were comminuted. As a result, crushed samples would be used for further analysis of oil and moisture content of *Camellia gauchowensis* Chang and *C. semiserrata* Chi seeds kernels, respectively.

### 3.2. NIRS Analysis of Camellia Seeds Kernels

The light absorption causes vibration and oscillation between atoms to make the change of light energy [30]. Therefore, the diverse spectrums in NIRS area indicated the differences in the moisture content, and the oil chemical composition and content for both *Camellia gauchowensis* Chang and *C. semiserrata* Chi seeds kernels.

#### 3.2.1. NIRS Analysis of *Camellia* Oil

The calibration models had good accuracy for the detection of *C. gauchowensis* Chang and *C. semiserrata* Chi kernels oil (Table 1 and Table 2). Some spectral variables, including irrelevant information and unreliable prediction, were removed by suitable algorithms [32] from all 110 samples by principal component analysis (PCA), in which the effective quantities of tested samples reached 106 for *C. gauchowensis* Chang and 104 for *C. semiserrata* Chi, respectively.

Table 1 exhibits the calibration statistics for oil content of *C. gauchowensis* Chang and *C. semiserrata* Chi kernels. For the 106 *C. gauchowensis* Chang samples, the maximal oil content was 68.43%, the minimum 23.85%, mean ± standard deviation (SD) 48.71 ± 8.94%, and coefficient of variation (CV) 0.18. For the 104 *C. semiserrata* Chi kernels, the maximum, minimum, mean ± SD, and CV of oil content were 71.08%, 31.71%, 58.37 ± 7.39%, and 0.13, respectively. The difference between Mean and Max (Min) was obvious, which meant the model could predict the validation set of samples well [7].

Different properties of samples have different spectrum. Therefore, Soxhlet method was used to treat the influence on the NIRS. In this study, the best spectrum pretreatment method was first derivation for the NIRS calibration model of oil content. The Rc, SEC, offset, bias, and slope were evaluated (Table 2). A good selected model should have high correlation coefficient of calibration (Rc) and low standard error of calibration (SEC) [33,34]. In our study, Rc values were high, 0.98 for *C. gauchowensis* Chang kernels oil and 0.95 for *C. semiserrata* Chi. Besides, SEC values were 1.57 for *C. gauchowensis* Chang kernels oil and 1.72 for *C. semiserrata* Chi, while SEP was 1.73 for *C. gauchowensis* Chang oil and 1.92 for *C. semiserrata* Chi. Results indicated that the SEC or SEP for *Camellia* oil in this study was relatively lower than sunflower (15.4–32.7 of SEP) [24] but higher than oilseed *Brassica* species (0.21–0.85 of SEC/SEP) [16], soybean (0.07–0.10 of SEP), and palm kernel (0.10–0.73 of SEP) [31]. The models’ accuracies for *C. gauchowensis* Chang and *C. semiserrata* Chi were both evaluated by offset (1.59 and 5.26, respectively), bias (1.99 × 10^−6^ and 1.26 × 10^−6^, respectively), and slope (0.97 and 0.91, respectively).

Typically, calibration models must be validated before application in practice [35]. Hence, the oil content by NIRS should be estimated with high correlation and good accuracy [36]. Partial least squares (PLS) regression was used to develop the quantitative model from cross-validation with different spectra pretreatments (data not shown). Table 1 shows the oil content of *C. gauchowensis* Chang and *C. semiserrata* Chi kernels in validation set. Besides the minimum and mean of *Camellia semiserrata* Chi, the data range of all calibration set covered the validation set. The maximal oil content of the 106 *C. gauchowensis* Chang samples in validation set was 56.82%, minimum 22.16%, mean ± SD 45.32 ± 7.57%, and CV 0.17. For the 104 *C. semiserrata* Chi kernels, the maximum, minimum, mean ± SD, and CV of oil content were 70.00%, 51.71%, 62.73 ± 4.38%, and 0.07, respectively.

The ratio of performance to deviation (PRD) indicates excellent validation accuracy when the value is more than 2.5; a value between 2.0 and 2.5 indicates approximate prediction is possible; and a value less than 1.5 indicates the prediction is poor [37]. In this study, RPD values were all above 2.5 (5.94 for *C. gauchowensis* Chang kernels oil and 4.92 for *C. semiserrata* Chi oil). The validation models of oil content for *C. gauchowensis* Chang and *C. semiserrata* Chi seeds kernels showed preferable accuracy.

**Figure 2:**
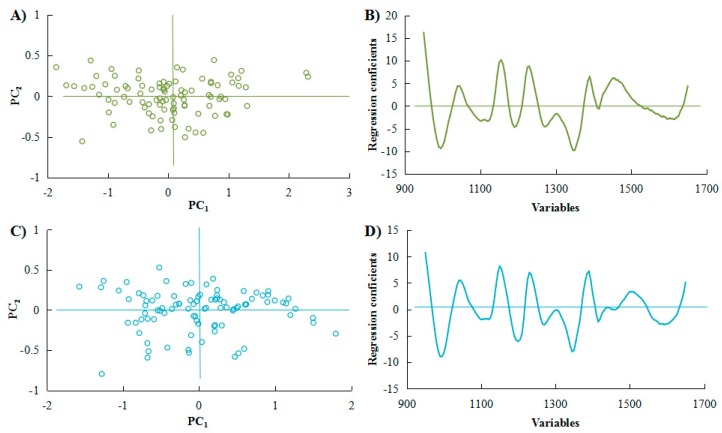
NIRS kernels oil content analysis of: *Camellia gauchowensis* Chang (**A**,**B**); and *C. semiserrata* Chi (**C**,**D**).

**Figure 3:**
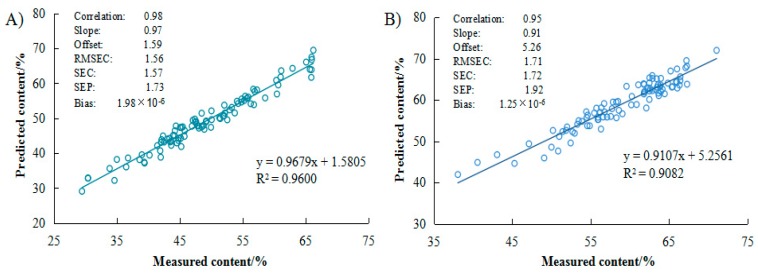
Scatter plots of measured versus predicted oil content of kernels for the PLS models by NIRS: (**A**) *Camellia gauchowensis* Chang; and (**B**) *C. semiserrata* Chi.

Figure 2A,C shows that the sample scores were uniformly distribution by PCA. After the PCs from 0 to 20 were analyzed by residual validation variance, the regression coefficients were ideal when the PCs were both selected at 5 (Figure 2B,D). In Figure 3, the linear relationships were established between predicted and measured values. The regression equations were *y* = 0.9679*x* + 1.5805 (*R*^2^ = 0.96) for *C. gauchowensis* Chang and *y* = 0.9107*x* + 5.2561 (*R*^2^ = 0.91) for *C. semiserrata* Chi, which was similar to the accuracy of rapeseed (*R*^2^ = 0.91–0.96) [33].

Thereby, the models by NIRS of *C. gauchowensis* Chang and *C. semiserrata* Chi seeds kernels were practicable to predict the oil contents.

#### 3.2.2. NIRS Analysis of *Camellia* Moisture

In Table 1, the maximum, minimum, mean ± SD, and CV of the moisture content of *C. gauchowensis* Chang samples in calibration set were 9.02%, 2.40%, 4.39 ± 1.08%, and 0.25, respectively. For the *C. semiserrata* Chi kernels, they were 6.37%, 2.32%, 3.49 ± 0.71%, 0.20, respectively. The oil content of *C. gauchowensis* Chang (48.71% of mean) was lower than *C. semiserrata* Chi (58.37% of mean); contrarily, the moisture content was higher (4.39% of *C. gauchowensis* Chang and 3.49% of *C. semiserrata* Chi, respectively). The lower moisture content is considered important for the specific application of *Camellia*, with regard to the economics of *Camellia* trading and quality-keeping to prevent the growth of microorganisms and mold spoilage [38]. *C. semiserrata* Chi seeds kernels had higher oil content and lower moisture compared with *C. gauchowensis* Chang, implying that *C. semiserrata* Chi was probably more suitable for cultivating, storage, processing, and application.

**Figure 4:**
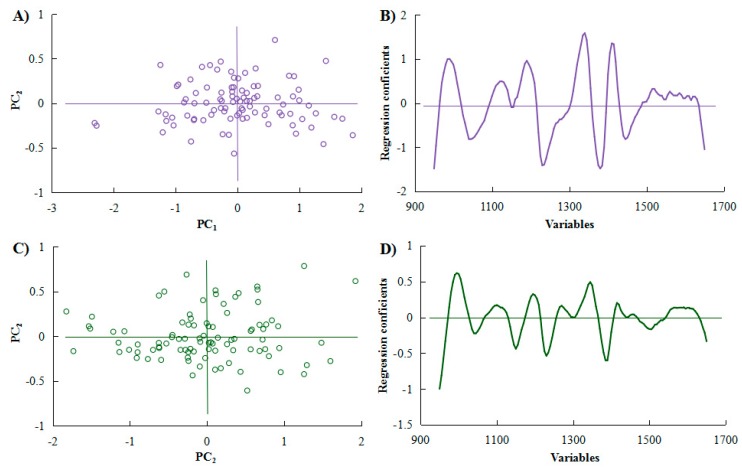
NIRS analysis kernel moisture content of: *Camellia gauchowensis* Chang (**A**,**B**); and *C. semiserrata* Chi (**C**,**D**).

**Figure 5:**
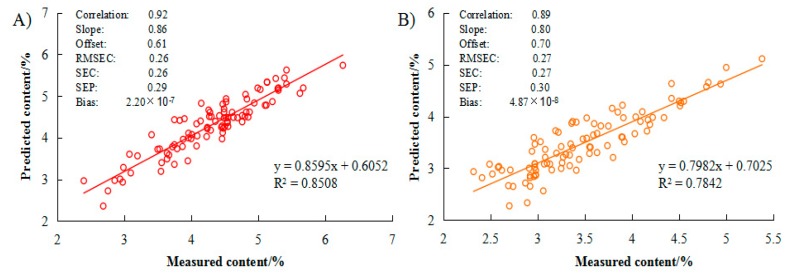
Scatter plots of measured versus predicted moisture content of: (**A**) *Camellia gauchowensis* Chang; and (**B**) *C. semiserrata* Chi kernels for the PLS models by NIRS.

The calibration model for *C. gauchowensis* Chang and *C. semiserrata* Chi seeds kernels moisture is shown in Figure 4. The scores showed uniform distribution by PCA in Figure 4A,C for 106 samples of *C. gauchowensis* Chang and 104 samples of *C. semiserrata* Chi. The relationship between validation variance and regression coefficients (Figure 4B,D, respectively) was analyzed to set up the prediction models (Figure 5); the calibration parameters of which are also shown in Table 2.

Rc value for moisture content of *C. gauchowensis* Chang seeds kernels was as high as 0.92, and 0.89 for *C. semiserrata* Chi. SEC for *C. gauchowensis* Chang and *C. semiserrata* Chi was 0.26 and 0.27, respectively, lower than sunflower (SEC of 1.6–3.2) [24]. The offset (0.61 and 0.70, respectively), bias (2.20 × 10^−7^ and4.87 × 10^−8^, respectively), and slope (0.86 and 0.80, respectively) values were all within the reasonable range for these two species.

For *C. gauchowensis* Chang and *C. semiserrata* Chi samples, the maximum (9.00 and 5.15, respectively), minimum (2.74 and 0.71, respectively), mean ± SD (4.62 ± 0.84% and 3.19 ± 0.84%, respectively), and CV (0.18 and 0.26, respectively) in validation set are shown in Table 1. Table 2 presents that SEP was 0.29 and 0.30, respectively, lower than black tea (SEP of 2.8–3.0) [38]. The RPD values (4.22 and 2.77, respectively) were both above 2.5. The regression equations were *y* = 0.8595*x* + 0.6052 (*R*^2^ = 0.85) for *C. gauchowensis* Chang (Figure 5A) and *y* = 0.7982*x* + 0.7025 (*R*^2^ = 0.78) for *C. semiserrata* Chi (Figure 5B), respectively. All results implied a good predictive ability of the calibration model for *C. gauchowensis* Chang and *C. semiserrata* Chi kernels moisture content.

## 4. Conclusions

This study demonstrated that NIRS was a powerful technique to predict oil and moisture content of *Camellia gauchowensis* Chang and *Camellia semiserrata* Chi seeds kernels. Principal components analysis (PCA) was used and the sample scores examined to detect outlying or unusual spectra [25]. Then, partial least squares (PLS), one of the most classical multivariate calibration methods, was obtained the best calibration models in this research. High coefficient of calibrations showed that NIRS analysis, a fast and effective method, could be applied in *Camellia* oil industry. This predictive model had good accuracy and sufficient credibility. Compared with traditional chemical measurement and analysis, NIRS possessed the advantage of obviously shortened detection time, mainly due to the practical calibration models. Based on the establishment of a relatively accurate calibration model, the efficient determination of oil and moisture content for large number of *C. gauchowensis* Chang and *C. semiserrata* Chi seeds kernels samples could be realized. It would be important with regard to the economics of *Camellia* trading and potentially impact the procurement price of two species oil.

## Figures and Tables

**Table 1:** Calibration and validation statics in NIRS models for the estimation of oil and moisture contents of *C. gauchowensis* Chang and *C. semiserrata* Chi kernels used in sets (n = 110).

Indicators	Seeds Kernels	Number	Statistics	Max ^1^/%	Min ^2^/%	Mean ± SD ^3^/%	CV ^4^
Oil	*Camellia gauchowensis* Chang	106	Calibration set	68.43	23.85	48.71 ± 8.94	0.18
			Validation set	56.82	22.16	45.32 ± 7.57	0.17
	*C. semiserrata* Chi	104	Calibration set	71.08	31.71	58.37 ± 7.39	0.13
			Validation set	70.00	51.71	62.73 ± 4.38	0.07
Moisture	*C. gauchowensis* Chang	106	Calibration set	9.02	2.40	4.39 ± 1.08	0.25
			Validation set	9.00	2.74	4.62 ± 0.84	0.18
	*C. semiserrata* Chi	104	Calibration set	6.37	2.32	3.49 ± 0.71	0.20
			Validation set	5.14	0.71	3.19 ± 0.84	0.26

Note: ^1^ Max, Maximum; ^2^ Min, Minimum; ^3^ SD, standard deviation; ^4^ CV, coefficient of variation.

**Table 2:** Calibration and validation model parameters of oil and moisture contents by NIRS.

Indicators	Seeds Kernels	Rc ^1^	SEC ^2^	SEP ^3^	Offset	Bias	Slop	RPD ^4^
Oil	*Camellia gauchowensis* Chang	0.98	1.57	1.73	1.59	1.99 × 10^−6^	0.97	5.94
	*C. semiserrata* Chi	0.95	1.72	1.92	5.26	1.26 × 10^−6^	0.91	4.92
Moisture	*C. gauchowensis* Chang	0.92	0.26	0.29	0.61	2.21 × 10^−7^	0.86	4.22
	*C. semiserrata* Chi	0.89	0.27	0.30	0.70	4.87 × 10^−8^	0.80	2.77

Note: ^1^ Rc, Correlation coefficients of calibration; ^2^ SEC, Standard error of calibration; ^3^ SEP, Standard error of prediction; ^4^ RPD, Ratio of performance to deviation (Standard deviation/SEP).
